# Adapting the myPlan safety app to respond to intimate partner violence for women in low and middle income country settings: app tailoring and randomized controlled trial protocol

**DOI:** 10.1186/s12889-020-08901-4

**Published:** 2020-05-29

**Authors:** Michele R. Decker, Shannon N. Wood, S. Rachel Kennedy, Zaynab Hameeduddin, Catherine Tallam, Irene Akumu, Irene Wanjiru, Ben Asira, Benjamin Omondi, James Case, Amber Clough, Richard Otieno, Morris Mwiti, Nancy Perrin, Nancy Glass

**Affiliations:** 1 Department of Population, Family and Reproductive Health, Johns Hopkins Bloomberg School of Public Health, Baltimore, MD 21205 USA; 2 Center for Public Health & Human Rights, Johns Hopkins Bloomberg School of Public Health, Baltimore, MD USA; 3 Johns Hopkins School of Nursing, Baltimore, USA; 4 Ujamaa-Africa, Mashinani Department, Nairobi, Kenya; 5 Department of International Health, Johns Hopkins Bloomberg School of Public Health, Baltimore, USA; 6 Center for Global Health, Johns Hopkins University, Baltimore, USA

**Keywords:** Intimate partner violence, Safety planning, Harm reduction

## Abstract

**Background:**

Intimate partner violence (IPV) is a leading threat to women’s health and safety globally. Women in abusive relationships make critical decisions about safety and harm reduction while weighing multiple competing priorities, such as safety of children, housing and employment. In many low- and middle-income countries (LMIC), IPV prevention and response services are limited and women lack access to safety planning resources. In high-resource settings, an interactive safety decision aid app (myPlan) has been found valuable in reducing decisional conflict and empowering women to take action in accordance with their safety priorities. This paper describes 1) the community-participatory formative process used to adapt the myPlan app content, interface, and implementation for the Kenya context, and 2) the randomized clinical trial study protocol for efficacy evaluation of myPlan Kenya.

**Methods:**

A community-participatory formative process engaged service providers and stakeholders, as well as IPV survivors for adaptation, followed by an in-depth pilot and final refinements. A randomized clinical trial design will then be used to determine efficacy of the myPlan Kenya app compared to standard care among women reporting IPV or fear of partner and living in an urban settlement. myPlan Kenya app provides and solicits information on a) relationship health; b) safety priorities; and c) severity of relationship violence. Based on the woman’s inputs, the evidence-based algorithm developed for myPlan Kenya generates a tailored safety plan. Outcome measures are assessed at baseline, immediate post-intervention, and 3-month post-baseline. Difference-in-differences analysis compares primary (e.g. safety preparedness, safety behavior, IPV), and secondary outcomes (e.g. resilience, mental health, service utilization, self-blame) across timepoints by group.

**Discussion:**

Formative phase revealed high feasibility and acceptability of a technology-based intervention for safety planning in this LMIC setting. This phase generated essential refinements to myPlan Kenya app readability, content and implementation, including increased visualization of messaging, and implementation via community health volunteers (CHVs). The resulting trial will be the first to evaluate efficacy of a community-partnered technology-based IPV intervention in a LMIC. Our adaptation process and trial results will inform researchers and interventionists to integrate multiple data sources to adapt IPV intervention content and interface in settings where technology-based interventions for IPV are novel and literacy is limited.

**Trial registration:**

Pan African Clinical Trial Registry approval received 25 April 2018 (PACTR201804003321122); retrospectively registered.

## Background

Globally, intimate partner violence (IPV) is a persistent threat to the health and wellbeing of women and girls. Eliminating IPV and other forms of gender inequity are targets of the Sustainable Development Goals [1]. Non-fatal consequences of IPV include poor mental, physical, and sexual health [2–10]. Over one third of female homicides globally are perpetrated by an intimate partner; and this proportion is on average six times higher for women relative to men [11]. In Kenya, national data from 2014 indicates that 39% percent of ever-married women age 15–49 have ever experienced spousal physical or sexual violence, relative to 9% of ever-married men in the same age group [12]. The prevalence of IPV is even higher in Nairobi’s informal settlements, and confers profound consequences to physical, sexual and mental health [13]. Social norms create a culture of IPV tolerance that challenges women’s ability to seek help or identify their experiences as abuse [14, 15]. Practices such as dowry payment for marriage contribute to strict social norms of gender inequity including the dominance of male partners on decisions related to sex, control, and authority in the relationship, regardless of the woman’s desires or preferences [16–19]. Comprehensive efforts to prevent and respond to IPV are urgently needed, particularly in low- and middle-income countries (LMICs), where IPV support resources can be scarce.

Safety planning is one of the most widely recommended interventions to prevent and respond to IPV. Disclosing abuse and obtaining support is beneficial [20–22], and can reduce post-traumatic stress [22], self-blame [23], and revictimization [24–26]. Too few survivors receive this support due to shame, self-blame, concern about stigmatization, and lack of knowledge about services [27–29]. Women also often face multiple, competing priorities in considering how to respond to IPV, with priorities including children, privacy, and financial security. Decision aids are an established tool in healthcare settings for providing information about options and clarifying personal priorities in order to make treatment decisions. The first decision aid to specifically address IPV-related decision-making and safety planning is *myPlan*, an interactive, personalized app developed by Johns Hopkins University (JHU) (www.myplanapp.org).

The myPlan interactive app provides education on healthy relationships, and solicits information about current intimate relationship, including risk of severe and lethal violence in the relationship and safety priorities to prompt recognition of risk and generate safety strategies tailored to women’s priorities, including referrals for support [30–32]. Key features of the app include the interactive “red flags” for unhealthy relationship and the Danger Assessment (DA), a measure of risk factors for severe and lethal violence in abusive relationships [33], with an instant score converted to danger level. An interactive visual aid enables prioritization of safety priorities (e.g. health and well-being of children, having resources, privacy) via systematic pair-wise comparison using a clickable “sliding bar” for the user to rank priorities. Based on input data, a personalized safety plan with relevant referral information is generated from the evidence-based algorithm designed for the app. As it is common for abusive partners to monitor survivors’ digital activities, myPlan safety features include: 1) a 4 digit security PIN for logging in; 2) instructions for entering a “dummy” PIN that takes the user to alternate content (in this context a cooking website was deemed most appropriate) if the app is discovered and they are forced to log in; and 3) access via web or downloadable app, allowing users to choose the safest method of access.

myPlan draws on elements of social cognitive theory [34], Dutton’s empowerment model [35] and trauma-informed care (TIC) [36, 37], which emphasize safety and empowerment through decision-making and healing. myPlan provides support for 1) defining healthy relationships, with descriptions of behaviors that are not healthy; 2) safety strategies, by helping women identify the severity of the violence and potential danger to self and family, 3) decision-making, via identifying safety priorities (e.g., privacy/confidentiality, children, feelings for partner) and thus reducing decisional conflict and enhancing safety preparedness, and 4) healing via validating messages to counter the culture of victim-blaming, further bolster resilience, and enable safety behavior and connection to both formal (e.g. hotline, health care providers, advocates) and informal (family/friends, co-workers) support services, thus ultimately enhancing health and reducing violence.

Women who have used myPlan have found it a unique opportunity to privately consider safety priorities, receive information about danger in relationships, and serve as an ongoing accessible resource for safety. In trials in high-resource settings, women that used myPlan reported reductions in decisional conflict about safety and increased use of helpful strategies that promoted health, safety, and well-being [38]. They were also more likely to leave an abusive relationship than those in the control group [38], though it is well-understood that leaving an abusive relationship is not always feasible or preferable, nor does it ensure safety. myPlan has been implemented in a range of high resource settings (e.g. USA, Canada, New Zealand, and Australia) with women of all ages [31, 39, 40].

Deploying this technology in LMICs requires formative work and collaboration with community partners and survivors to determine feasibility and acceptability of the content and delivery as well as rigorous evaluation. To advance the global priority of evidence-based interventions for women in LMICs that prevent IPV and mitigate its health impact, and cross-validate an app that have been developed in other settings, our team conducted extensive tailoring of the myPlan app for a LMIC setting (Nairobi, Kenya). This paper describes 1) the adaptation process, and 2) the study protocol for the efficacy evaluation of myPlan Kenya. This paper describes the contextual considerations necessary to ensure transferability of the myPlan app to IPV survivors in urban informal settlements of Nairobi, while maintaining consistency with intervention components found effective in other settings. The protocol is approved by the Johns Hopkins Bloomberg School of Public Health Institutional Review Board (IRB) and the Kenya National Commission for Science Technology and Innovation, and registered with the Pan African Clinical Trial Registry as PACTR201804003321122. This study did not assemble an independent data monitoring committee because the follow-up duration was relatively short, and because the majority of study enrollment was completed prior to obtaining follow-up endpoints. Interim results were reviewed at regular intervals by the investigative team; specifically the team reviewed IPV prevalence and mental health indicators.

### Community-partnered participatory research & study setting

All research was conducted in close collaboration with Ujamaa-Africa, a non-governmental organization (NGO) based in Nairobi, Kenya focused on violence prevention and response. The Mashinani department of Ujamaa provides economic empowerment to IPV survivors via support groups coupled with microfinance loans, affording the team unique insight into local IPV dynamics. The six-member Mashinani team led the implementation of the formative phase and randomized controlled trial (RCT), in close collaboration with the Johns Hopkins University (JHU) research team.

This study was conducted in three informal settlements in Nairobi, Kenya: Korogocho/ Kariobangi, Dandora, and Huruma/Mathare. The field office was the secure Mashinani office headquarters in Kariobangi North. Two other informal settlements, Dandora and Huruma/Mathare, were chosen for their geographic and economic diversity, as well as the established rapport of Mashinani in these communities.

## Formative phase to tailor app content and Interface for feasibility, Relevance & Acceptability

The formative phase was conducted from May to December of 2017. Community stakeholders and violence prevention/response practitioners were engaged for key informant discussions (KIDs), and adult (18 years or older) IPV survivors were recruited for focus group discussions (FGDs), all recruited via purposive, community-based sampling in collaboration with Ujamaa Mashinani team.

### Key informant discussion and IPV survivor focus group discussion data collection

Key informants are community health volunteers (CHVs), chiefs or leaders, and other service providers working with IPV survivors in the targeted settlements. Key informants were identified via engagement with local IPV organizations. Key informants were eligible if they were 18 years or older, had experience working directly with female IPV survivors in Nairobi and provided informed consent*.* FGDs were conducted with IPV survivors. FGD participants were recruited primarily through word-of-mouth, supported by flyers and presentations at local organizations and chief offices. Eligible women were 18 years or older, had experienced physical, sexual, or emotional violence by a current or former partner in the past 12 months. Recruitment continued until repetition of themes (saturation) was achieved [41].

KIDs and FGDs with IPV survivors lasted approximately 90 min and were conducted in private locations. Data collection was conducted by a team of three female Kenyan interviewers in English and Swahili. Interviewers received two weeks of qualitative research training prior to study implementation, including mock sessions. On-site support from a seasoned qualitative researcher was available.

Following a semi-structured guide, the KID and FGDs were designed to inform the myPlan Kenya app content, interface, and implementation. Content-related probes centered on women’s experiences of violence, recommended and utilized harm reduction and safety strategies, community norms and beliefs about IPV, and availability of community resources for IPV survivors. Interface and implementation-related probes centered on feasibility, design, and use of smartphones for delivering the app with women in the local area. Mock designs of the app were color-printed on large, laminated pages to appear as screen shots of each app component. They were used to solicit feedback from participants on appearance, content and user experience. Key informants were additionally asked about IPV support services; study team members met with organizations recommended during KIDs to understand scope of services and assess fit as a participant referral within the app, and during the subsequent RCT referral.

### Key informant discussion participants

A total of 16 key informants participated in seven semi-structured KIDs of three to four participants. Two key informants completed individual discussions due to logistical constraints. KID participants were primarily female (*n* = 15 female, *n* = 3 male) and averaged 9.7 years of work experience with IPV survivors. Roles included: four social workers, five CHVs, one chief, two psychosocial therapists or counselors, two advocates focused on gender justice, and four NGO employees who worked directly with survivors.

### IPV survivor participants in focus group discussions

After informed consent, the majority of the survivors participated in one of six semi-structured FGDs, with an average of eight per group. One survivor opted for an individual interview for privacy. FGD participants had a mean age of 32.3 (SD = 12.6). Approximately 30% identified as Kikuyu and Luhya, with fewer identifying as Luo (14.3%), Kamba (14.3%), Borana (4.1%), and Meru (4.1%). Nearly 80% of women were in a current relationship and had on average two children (SD = 1.6). Almost half (49.0%) of survivors reported past month physical violence and 36.7% reported past month sexual violence.

### Qualitative analysis & triangulation of input from multiple sources

All qualitative data were audio-recorded, transcribed, translated verbatim from Swahili to English, and quality checked by a team of six transcribers. Three Masters-level research assistants coded all interviews in Atlast.ti.1.6 using inductive thematic analysis. To improve internal consistency, multiple team member checks and revisions were performed. Mashinani staff engaged in data interpretation via participatory triangulation of data facilitated by weekly team debriefs with the on-site field researcher, lead investigators, and Mashinani staff. Field notes were maintained and referenced for triangulation of data. Triangulation of results across IPV stakeholders and survivors occurred via convergence matrices which displayed key findings across data sources to identify areas of agreement, partial agreement, silence, or dissonance [42].

### Pilot testing of the refined myPlan Kenya app

The myPlan Kenya app was adapted based on input gleaned from IPV survivors and stakeholders as described above. In cases of dissonance, silence, or partial agreement, recommendations prioritized the input of IPV survivors. All content was translated into Swahili and reviewed by native speakers for comprehension. The resulting myPlan Kenya app was pilot tested with 18 IPV survivors in December 2017. Participants tested the app and completed an in-depth interview with trained research assistant to clarify feasibility and acceptability. Pilot testing and interviews lasted approximately 90 min and were conducted one-on-one in a private setting. Structured notes were taken to inform updated convergence matrices and finalize intervention format and implementation.

### Summary of intervention app adaptations and their rationales (Table 1)

#### Content

The primary content adaptations centered on safety strategies to reflect women’s realities, including the social pressures for partnership preservation and childbearing. The formative phase identified domains of safety strategies that required development and refinement. Primary examples included child safety strategies in the moment of conflict, as well as options for women to temporarily leave the home and visit family to stay safe or gain leverage for partner changing his behavior. The myPlan app does not recommend staying or leaving a given relationship but rather provides tailored strategies for safety that were adjusted as recommended by survivors and key informants to emphasize harm reduction to “stay safely” rather than leave the relationship, in response to the strong social stigma against relationship termination in this setting.

**Table 1:** Summary of formative phase results and corresponding adaptations to content, interface and planned implementation strategy

		**Source(s)**			
**Qualitative Finding/Rationale**	**Intervention Adaptation**	**FGD**	**KID**	**Intervention adaptation leadership field team** **(Mashinani)**	**Pilot**
**App Content**					
Leaving relationship safely not considered feasible in this setting	Emphasis on harm reduction strategies for “staying safely” in relationship	X			
Strong local preference and pressure to endure violence		X			
Cultural norms and relationship dynamics with in-laws and natal families	Development of temporary leaving strategies to natal family home and in-law home	X		X	
Permanent separation from partner not considered feasible in this context		X		X	
Preference for short-term options that do not compromise family status in the community		X			
Parental stress over children exposed to violence	Addition of age-specific child safety strategies, and sexual/reproductive health safety strategies	X			
Distinct cultural and tribal norms around child custody		X	X	X	
Concern for risks and harms incurred during pregnancy		X			
Pressure specific to childbearing and care for children		X		X	
Fear of community stigma around violence	Modification of ‘priorities’, ‘healthy relationships’, and ‘harmful beliefs about abuse’ section of the app to reflect those in this context	X			
Pressure specific to childbearing and care for children		X		X	
Common beliefs and myths regarding healthy and unhealthy relationships in this context		X	X	X	
Participant challenges interpreting the danger assessment (homicide risk assessment)	Danger assessment result presented to participants as a binary (increased danger vs. variable danger) for simplicity				X
**App Interface**					
Time needed to review and complete each app component app negatively impacted feasibility and engagement	Components of app re-organized and shortened where possible			X	X
Preference to use sliding scale for ordering personal priorities	Rank system removed and replaced with sliding scale for ordering personal priorities				X
Wide range of literacy including low literacy	Inclusion of graphics and short animations			X	X
**App Implementation**					
Women’s familiarity with apps is limited though growing	Community health volunteers (CHVs) trained to facilitate app implementation		X	X	X
Women may lack confidential space to use the phone without arousing suspicion		X			
Phone-sharing is common		X			
Quality of phones and ability to support apps is limited in some areas, though expanding			X	X	

#### Interface and implementation

Participants suggested CHVs could help orient women to the app to maximize feasibility, acceptance, and impact. This recommendation was adopted for the resulting RCT protocol, which engaged CHVs for recruitment, data collection, implementation of intervention app and control condition, interpretation of results, and dissemination.

Pilot test feedback centered primarily on app format and implementation. The initial refined app was too lengthy, taking 1.5–2 h to complete. Participants recommended streamlining the app content for brevity, to allow for full engagement with each component. Graphics and animated videos were recommended for readability and interactive support, and were developed and finalized with extensive input from the Mashinani team. Following the pilot phase feedback, safety strategies were positioned earlier in the app process for accessibility. Participants also advised the content of the safety strategies be more tailored to women’s personal circumstances, particularly regarding child safety and leaving strategies.

## Randomized controlled trial methods

### Design

This trial is designed as a randomized, controlled, participant-blinded superiority trial with two parallel groups (1:1 allocation ratio), with primary end points of safety preparedness, decisional conflict (e.g. clarity of values, knowledge), use and helpfulness of safety strategies, and IPV.

We hypothesized women in the myPlan Kenya safety decision app group would have increased safety preparedness, reduced decisional conflict about safety priorities, increased helpfulness and use of safety behaviors, and reduced IPV at three-months post-baseline in comparison to the control group.

### Data collector training

Local CHVs were hired to supplement the Mashinani team for data collection and intervention implementation. The data collection team underwent a month-long training process that covered data collection procedures, app content and procedures, tablet orientation for delivering the myPlan Kenya app, survey measures, and ethical training, including that specific to research on gender-based violence [43], with mock data collection and intervention sessions.

### Recruitment of participants

Eligible women were ages 18–35 years-old, had experienced physical or sexual IPV, or reported being afraid of their partner in the last three months, resided in Korogocho/Kariobangi, Dandora, or Huruma/Mathare with no plans to move in the next six months, and spoke English or Swahili language. Community-based recruitment occurred through flyers, community presentations, and word-of-mouth. This passive recruitment method is a sensitive way to recruit IPV survivors, allowing them the opportunity to decide on study participation without feeling pressured.

**Fig. 1:**
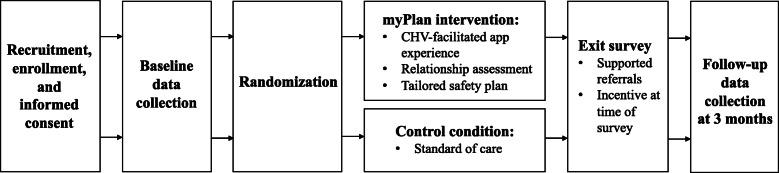
Study process diagram, including time points for assessment and enrollment

Local CHVs aided in raising awareness about the study and referring potential participants. Recruitment events held specifically with CHVs informed them of the study objectives and sought feedback on recruiting eligible women. CHVs then worked in tandem with the recruitment team to refer potential participants. Study participants were also offered recruitment flyers to notify others about the study. Recruitment continued until adequate sample size achieved in each of the three sites with an enrollment goal of 350 women. The study process is outlined in Fig. 1.

### Informed consent and enrollment

Participants contacted study staff by telephone or in person for more information about the study and pre-screening. Research staff clarified the purpose and parameters of the study, and eligibility criteria. Eligible participants were then invited to their preferred site for in-person data collection.

On-site eligibility confirmation via tablet occurred in private immediately prior to consent. Age and residence criteria were confirmed via single questions. Recent IPV or partner-related fear criteria were assessed via three items with “yes” to any satisfying eligibility criteria: 1) “Is there something about your partner that makes you feel unsafe or threatened?”; 2) “In the past 3 months, did your partner hit, punch, throw, slap, or kick you?” and 3) “In the past 3 months, did your partner ever force or pressure you to have sex when you didn’t want to?”

An oral consent process maximized participant confidentiality. To verify and document consent, the data collector initialed and dated the consent form saved within the tablet by study ID.

### Randomization to intervention or control condition

Following tablet-based baseline survey data collection, women were randomized to either the control or intervention arm within the app itself, stratified by study site (1:1 allocation); following randomization participants were seamlessly directed to either the control or the intervention app. The tablet further eased data collection given recording of all consent, survey data, and app measures, as well as analytics on time-to-completion and missingness of data. Intervention participation took approximately 30–45 min, whereas the control condition was limited to 10–20 min.

### Blinding

All participants were blinded. Study staff aiding participants with the app were necessarily nonblinded; all other study staff, including those involved in recruitment, enrollment/consent, warm referrals, and data monitoring, were blinded to intervention status.

### Baseline data collection

Survey data collection preceded randomization and took approximately 30–45 min. Interviewer-facilitated survey data collection and app assistance was recommended in the formative phase to maximize comprehension and interest. While some participants preferred to complete the survey almost entirely on their own, others made use of the available assistance from trained study team members. All data collection was recorded on a tablet to allow the use of on-screen buttons and clickable sliding bars, as well as audio visual to enhance interaction.

## Measures

### Primary outcomes

Primary outcomes include safety preparedness, decisional conflict, use and helpfulness of safety strategies, and IPV.

A 10-item scale assessed safety preparedness immediately following completion of intervention or control condition. Items focused on the value of the reviewed material for readiness in decision-making and weighing of risk/benefits, e.g., “to what extent did the material prepare you to make safety decisions,” with participants indicating preparedness on a Likert scale (Responses: 0-Not At All to 4-A Great Deal; Range: 0–40; modelled continuously).

A 12-item adaptation of the Decisional Conflict Scale (DCS), which discriminates those who make decisions and those who delay [44], asked participants to rate their knowledge, clarity and confidence in safety options and risk/benefits of potential options on a Likert scale [30] (Responses: 0-Not At All to 4-A Great Deal; Range: 0–48; modelled continuously).

Use of safety strategies was assessed through five-item index of strategies specific to content area covered in the app; the index was adapted from previous myPlan trials and formative work in the U.S. [26, 30, 45] Specifically, participants were asked “In the past three months, have you: 1) Left the house temporarily to put space between you and your partner; 2) Developed an emergency signal to use with others when you felt you were in danger; 3) Put a plan into place for how to keep your children safe; 4) Begun saving money to build self-sufficiency; and 5) Been part of a loan savings group in order to improve your family’s financial situation. If used, participants were then asked to rate the helpfulness of the strategy on a Likert scale (Responses: 1-Not Helpful to 5-Very Helpful). Responses generated two summary metrics: use of safety strategies reflecting number of strategies used (Range: 0–5), and helpfulness of strategies used, reflecting both use and helpfulness by dividing summed helpfulness by number of strategies used (Range: 0-Never Used to 5-Used and Found Helpful).

Longer-term outcomes focused on IPV experiences. Physical and sexual IPV were assessed through the short forms of the Revised Conflicts and Tactics Scale (CTS-2) [46]. For each of ten violent behaviors, women were asked frequency of occurrence in the past 3 months: Never (0), 1–2 times (1), 3+ times (2), and scores summed across behaviors (Range: 0–20); IPV will be examined as both continuous and binary for physical IPV only, sexual IPV only, and combined physical and sexual IPV. The Women’s Experiences of Abuse (WEB) Scale [47] captured the lived experience of abuse (Responses: 1-Agree Strongly to 6-Disagree Strongly; Range: 10–60). Reproductive coercion was assessed via existing 9-item measure [48, 49] deemed appropriate based on formative research and summarized into a binary measure indicative of experience/no experience. All IPV assessments used behavioral definitions in accordance with best practices [43].

### Secondary outcomes

Secondary outcomes include resilience, relationship quality, depression, consideration and seeking of IPV support services, self-blame, recognition of abuse, self-efficacy, and danger score.

Resilience was assessed with the ten-item Connor-Davidson Scale [50], which has been validated in East Africa [51] (Responses: 0-Not True At All to 4-True Nearly All the Time; Range: 0–40; modelled continuously).

Relationship quality was assessed with four items adapted from the CTS-2 negotiation sub-scale [46] deemed most applicable by the field team. Respondents rated frequency of respectful relationship experiences on a 4-response Likert Scale (Responses: 0-Never to 3-Almost Always; Range: 0–12). Responses were summed and modelled continuously.

Depression was assessed through the 10-item Center for Epidemiologic Studies Depression Scale (CESD-R; Responses: 0-Not At All/Less Than 1 Day to 3-Nearly Every Day; Range: 0–30) [52, 53] and modelled both continuously and dichotomously with a score > =10 indicating clinical depression.

Support service consideration and use, respectively, were assessed via a select-multiple item asking “In the past three months, have you considered seeking any of the following for partner-related violence or fears?“ [30, 45] Multi-select options included health, emergency, police/chief, legal, crisis hotline, counseling, housing, and children, based on previous myPlan trials and services identified during the formative phase. Participants endorsing having considered a given service were subsequently asked if they had sought that service. The outcome was handled dichotomously overall and for individual response; per item, a positive indication to any response was considered positive “considering” or “seeking.”

Self-blame was assessed through a 4-item adaptation of the characterological sub-scale of the Sexual Victimization Attributions Measure (SVAM) [54]. Frequency of self-doubt and blame was measured on a 5-response Likert scale (Responses: 0-Never to 4-Almost Always), and summed for a continuous metric (Range: 0–12).

Six recognition of abuse items were adapted from the Abusive Behaviors Scale [49, 55]. Items asked participants to consider six abuse behaviors on a 5-point Likert scale from 0 (Not Abusive at All) to 4 (Very Abusive). Items were summed and modelled continuously (Range: 0–24).

The 6-item Generalized Self-Efficacy Scale [56] includes broad statements about confidence, goalsetting, and coping (Responses: 1-Not True At All to 4-Exactly True; Range: 6–24). A 4-item Self-Efficacy for Safety in Relationship Scale [31] specifically examines confidence in relationship safety (Responses: 1-Strongly Disagree to 5-Strongly Agree; Range: 4–20). Both measures were summed for continuous modelling.

A 20-item Danger Assessment (DA) Scale [33] assessed homicide risk factors (Range 0–20). Items were only asked of intervention participants within the myPlan Kenya app.

### Description of the myPlan Kenya intervention app

The intervention app was CHV-administered based on formative phase input that suggested women would prefer to use the app at least once with a supportive volunteer or provider before using it on their own. The myPlan Kenya app included the following sections: Healthy Relationships, My Relationship, Red Flags, My Safety, My Priorities, My Plan, About Violence, Harmful Beliefs About Abuse, and Resources. Healthy Relationships is an educational component to help women think about their relationship and what constitutes a healthy relationship within their context. My Relationship gathers information about their relationship to help tailor safety strategies. Red Flags helps women understand and assess warning signs of abuse within their relationship. At the end of the section, women are given a healthy relationship score (healthy, unhealthy, vs. unhealthy/unsafe) based on their responses. Safety strategies are also tailored to these individual items of abuse. My Safety is a danger assessement [33] consisting of validated risk factors for repeat violence and severe/lethal IPV, such as an increase in frequency/severity of violence, controlling behaviors, and weapon use. An instant danger assessment score is computed and converted to a level of danger (increased vs. variable) to prompt action for women at high levels of danger. The My Priorities section is an interactive visual aid that uses a clickable “sliding bar” to allow the participant to make pairwise comparisons to weigh priorities including dignity and respect, feelings for partner, health, and children. These priorities are combined mathematically to generate priority weights that are reviewed by users before moving on to the My Plan/safety plan section. The My Plan section is populated based on data supplied in the previous sections. For example, for women indicating children as a priority, the personalized safety plan messages include safety strategies specific to managing children in situations of violence. The safety plan is followed by supplemental information About Violence, Harmful Beliefs that may hinder their plan, and relevant Resources.

### Intervention Fidelity

Comprehensive training for the entire study team emphasized delivery of identical procedures for intervention and control participants. Randomization to either intervention or control app was completed automatically on the tablet to maximize fidelity and minimize error. Once a participant was randomized, the tablet did not allow switching of intervention condition. The app-based format maximized fidelity in intervention implementation. Intervention fidelity was further monitored by both JHU and Mashinani team members through an online tracker to ensure 1:1 enrollment and completion of each condition. Referrals were provided by a designated referral team to ensure that both intervention and control participants received the same standard set of referrals, regardless of experience disclosed during the survey or intervention condition.

### Description of the control condition

Comparable to the standard of care, the control condition consisted of a standard set of safety strategies with an emphasis on referral resources. For comparability to the intervention arm, content was delivered in the form of an app, staff were present to assist women in understanding material, and participants given the option to return to the study office to review.

### Post-intervention assessment

Following receipt of intervention or control app content, participants completed a brief exit survey facilitated by the tablet. They completed a brief upset screener. All participants were offered a small grocery item as appreciation for their participation, as well as transport reimbursement.

### Referrals

All participants were offered referrals for IPV-related medical, psychosocial, or economic support. Those interested in referrals were directed to a Referral Lead; Referral Leads were blinded to intervention status and separate from the data collectors. Participants were offered contact information and the option for a “warm” facilitated referral to reduce barriers to care for participants in both conditions. Participants had the option to return to the study center to review their safety plan and referral list prior to three-month follow-up visit.

### 3-month follow-up data collection and retention

**Fig. 2:**
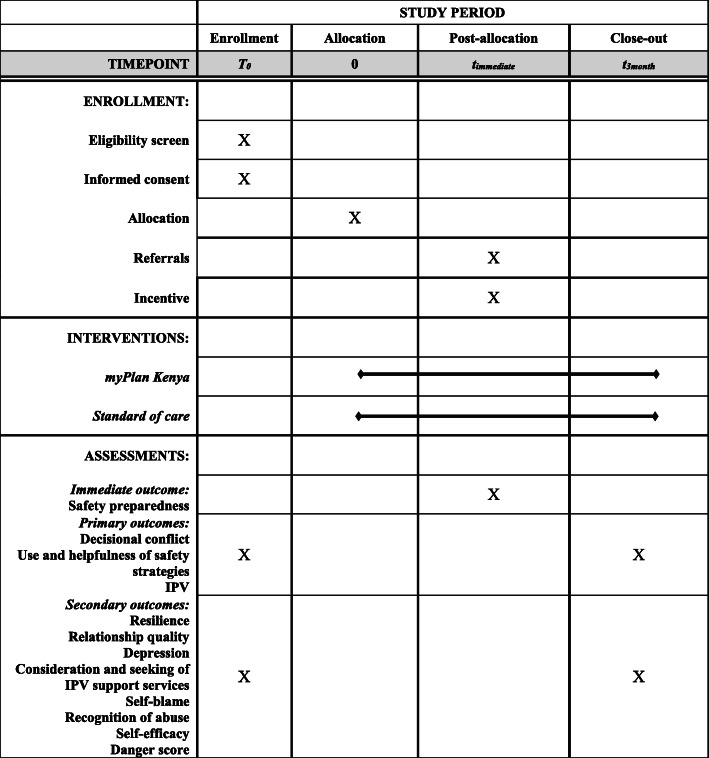
Schedule of enrollment, interventions, and assessments for myPlan Kenya

Participants were contacted for 3-month follow-up data collection at their original study office. Following refresher consent, survey data collection procedures were identical to those at baseline, specifically tablet-based data collection in English or Swahili language, lasting approximately 30–45 min, and with provision of resource referrals, transportation remuneration, small grocery item, and universal upset screener (Fig. 2). Intervention arm participants were also given the option for an in-depth interview at a later time period. Control participants were given the option to complete the myPlan Kenya app following survey data collection. The eligibility window for follow-up data collection began one week prior to the three-month anniversary of baseline through one month afterwards. A participant could be called up to five times; if such calls proved unsuccessful the research team attempted to reach her via her two provided alternate contacts. Participants were deemed ineligible for follow-up if they had moved out of the study area or if they were temporarily traveling and would not return by the end of the follow-up period.

### In-depth interview

Intervention participants who completed the baseline and follow-up surveys were eligible for a 30-min in-depth interview (IDI). Purposive sampling ensured a range of violence experiences and study sites (*n* = 30). Semi-structured interview guides focused on participant’s experiences with and recommendations for the intervention. Interviews were audiorecorded, transcribed verbatim, and translated to English language for analysis.

## Risk & safety

### Participant safety

Steps to enhance participant safety and confidentiality included baseline and refresher oral informed consent. At each session, a data collector explained procedures, answered questions, and signed as a witness to oral consent. All consent, survey data collection, app, and referrals were conducted individually in a private setting at the respective study site office.

All field and research team members completed human subjects and ethical training prior to study implementation. Formative and RCT training emphasized ethics specific to violence research. Precautions included skipping questions as necessary, monitoring reactions, and procedures for pausing and concluding interviews in case of severe upset or other adverse events.

### Study team safety

Study team precautions comprised conducting data collection during daylight hours, traveling to recruitment and data collection events in pairs, and using concealed bags for study material storage.

Buy-in from local communities including local chiefs was essential to success and safety of participants and staff. All local chiefs were contacted during the formative phase to introduce the app and study procedures, and solicit feedback. As part of recruitment, community presentations such as Parent-Teacher Association meetings, served to engage local community members in the study and solicit input.

### Contingency planning & monitoring for potential challenges

Throughout training, data collectors were given ample opportunity to contingency plan for a wide array of potential adverse events, in line with best practices for violence research [43]. Provisions were made to ensure immediate contact with the research team for swift response and timely IRB notification for any potential adverse events.

Twice weekly calls with the JHU team, and research support presence, including an on-site research fellow, throughout data collection facilitated field communication, protocol fidelity, monitoring for potential adverse events, and technical support. Technology issues were identified and resolved in real time, facilitated by online reports with screenshots to enable rapid troubleshooting. Over the seven-month data collection period, three site visits were conducted for the purposes of auditing data collection processes and documents, and field implementation observation. Extensive field notes submitted daily monitored the risk for social harm and adverse events, so as to enable rapid team communication and response.

### Data privacy measures

Identifying information to support retention included name (or pseudonym), phone number, and the names and phone numbers of two alternative contacts. All identifiers were stored in a separate password-protected database and only available to the recruitment team for follow-up calls. Survey data, app data and subsequent downloads were stored on a secure cloud server, as were IDI audio and transcripts.

## Data analyses

Prior to analyses, data quality will be examined through descriptive statistics, including tabulations and distributions to assess missingness. Imputation will be used for variables with missingness < 10%. Differences in intervention and control groups will be assessed for outcomes and potential confounding variables using Chi-square tests (categorical variables) and t-tests/ANOVA (continuous variables). Significance will be set at 5%. All analyses will account for site and within-person clustering. Attrition analysis comparing key demographics and baseline indicators across those lost-to-follow up with those retained will identify potential biases in the final retained sample.

### Intervention efficacy

All analyses will be performed using intent-to-treat principles. Primary analysis uses a differences-in-differences approach to compare differences between baseline and 3-month follow-up by intervention group. Random effects logistic and linear regression models will be created, including study arm, pre/post status and their interaction term (study arm*pre/post status). Interaction terms significant at *p*-value < 0.05 will indicate a significant intervention effect.

### Moderation analyses

Sub-aims of this study include examination of mediating pathways to change, and effect modification, i.e., sub-populations for whom the app may be more efficacious. Main effects analyses stratified by intervention group will explore effects underpinning the interaction term. Further stratified analyses will examine key moderators including demographic/background characteristics, violence severity, resilience, and violence-related help-seeking. Effect sizes will be compared between the groups, as the stratified analyses will not be fully powered.

### Statistical power

As decisional conflict estimates were unavailable for target population, the study powers on IPV; 2014 Kenya national data estimate 39% lifetime IPV prevalence for women [12]. Setting alpha = 0.05 and power to .80, a study with 175 intervention participants and 175 controls will find a significant difference between the groups if 25% of women experience violence in the intervention group compared to 39% in the control group [57]. If attrition rate is 15%, we will be able to detect significant difference if 24% of the intervention group experiences IPV.

## Discussion

Our extensive formative phase revealed high feasibility and acceptability of community-partnered technology-based safety planning intervention within Nairobi’s informal settlements, where the high prevalence and health impact of IPV has garnered calls for prevention and response [13]. Moreover it generated necessary refinements to ensure successful implementation that builds on the strengths of this community. Context-specific app refinements included app simplicity, readability and content, particularly layout changes to allow increased visualization of messaging and easier access to safety strategies; these refinements may aid in adaptations to additional settings. The planned RCT has the potential to significantly advance the science of IPV prevention and response in LMIC settings, where the evidence base on mHealth interventions for IPV is in its infancy [58].

In contrast with the higher-income settings in which myPlan was initially developed for confidential, anonymous access, women in our formative phase expressed a preference for facilitated administration of myPlan Kenya. Accordingly, our team trained CHVs to support women in using the app for the first time. An unanticipated but highly relevant outgrowth of the formative phase is that the intervention app is poised to function as an important job aid for CHVs and the other lay professionals that serve as important sources of informal support in global health systems. CHVs’ dual roles as community members and informal leaders [59] render them trusted confidants and sources of information on a range of health topics [59–66]. CHVs and other lay providers are increasingly benefitting from job aids [59], including mobile technology [67] and algorithm-based apps that can enhance decision-making [68]. This app could further be integrated into local professional services, including medical, counseling, and legal, to help disentangle women’s priorities when facing complex decisions.

The impact of the resulting trial, and the scale-up of this approach if found effective, is significant in that mobile connectivity of Kenya is almost at saturation; cell phone access now exceeds 94% in urban areas [12]. This strategy ensures nearly universal access for our target population. Study results will be presented to participating partners, key stakeholders, and the general public health community through dissemination forums, development of lay reports, and peer reviewed publications. The resulting app will be available in both downloadable mobile and web-based versions to maximize options in safe and secure access. To our knowledge myPlan Kenya is the first algorithm-based safety intervention app available in this setting that responds directly to the decisional conflicts faced by women experiencing IPV, and provides tailored safety strategies for responding to partner violence. We believe this will be the first experimental study to evaluate its efficacy in a LMIC, where IPV prevention and response needs are high and available services are often limited.

## Data Availability

The data used during the current study are available from the corresponding author on reasonable request.

## Abbreviations

CESD-R: Center for Epidemiologic Studies Depression Scale
CHV: Community health volunteers
CTS-2: Revised Conflicts and Tactics Scale
DA: Danger Assessment
DCS: Decisional Conflict Scale
FGD: Focus group discussions
IDI: In-depth interview
IPV: Intimate partner violence
IRB: Institutional Review Board
JHU: Johns Hopkins University
KID: Key informant discussions
LMIC: Low and middle-income country
NGO: Non-governmental organization
RCT: Randomized controlled trial
SVAM: Sexual Victimization Attributions Measure
TIC: Trauma-informed care
WEB: Women’s Experiences of Abuse Scale

## References

[1] UN. Sustainable Development Goal 5: Achieve gender equality and empower all women and girls. https://www.un.org/sustainabledevelopment/gender-equality/. Published 2016. Accessed.

[2] WHO (2005). WHO multi-country study on women’s health and domestic violence against women: summary report of initial results on prevalence, health outcomes and women’s responses.

[3] Ellsberg M, Jansen HA, Heise L (2008). Intimate partner violence and women's physical and mental health in the WHO multi-country study on women's health and domestic violence: an observational study. Lancet..

[4] Glass N, Fredland N, Campbell J, Yonas M, Sharps P, Kub J (2003). Adolescent dating violence: prevalence, risk factors, health outcomes, and implications for clinical practice. J Obstet Gynecol Neonatal Nurs.

[5] Lucea MB, Francis L, Sabri B, Campbell JC, Campbell DW (2012). Disordered eating among African American and African Caribbean women: the influence of intimate partner violence, depression, and PTSD. Issues Ment Health Nurs.

[6] Decker MR, Silverman JG, Raj A (2005). Dating violence and sexually transmitted disease/HIV testing and diagnosis among adolescent females. Pediatrics..

[7] Campbell JC (2002). Health consequences of intimate partner violence. Lancet..

[8] Lang DL, Sales JM, Salazar LF (2011). Rape victimization and high risk sexual behaviors: longitudinal study of african-american adolescent females. West J Emerg Med.

[9] Koenig MA, Zablotska I, Lutalo T, Nalugoda F, Wagman J, Gray R (2004). Coerced first intercourse and reproductive health among adolescent women in Rakai, Uganda. Int Fam Plan Perspect.

[10] Maharaj P, Munthree C (2007). Coerced first sexual intercourse and selected reproductive health outcomes among young women in KwaZulu-Natal, South Africa. J Biosoc Sci.

[11] Stockl H, Devries K, Rotstein A (2013). The global prevalence of intimate partner homicide: a systematic review. Lancet..

[12] Kenya National Bureau of Statistics, Ministry of Health/Kenya, National AIDS Control Council/Kenya, Kenya Medical Research Institute, National Council for Population and Development/Kenya, and the DHS Program/ICF International. Kenya Demographic and Health Survey 2014. Rockville: Kenya; 2015.

[13] Winter SC, Obara LM, McMahon S (2020). Intimate partner violence: a key correlate of women's physical and mental health in informal settlements in Nairobi, Kenya. PLoS One.

[14] Gillum TL, Doucette M, Mwanza M, Munala L (2018). Exploring Kenyan Women’s perceptions of intimate partner violence. J Interpers Violence.

[15] Maseno L, Kilonzo SM (2011). Engendering development: demystifying patriarchy and its effects on women in rural Kenya. Int J Sociol Anthropol.

[16] Black J (2017). Sex, abortion, domestic violence and other unmentionables: orthodox Christian youth in Kenya and windows into their attitudes about sex. Religions..

[17] Lowes S, Nunn N. Bride price and the wellbeing of women. WIDER Working paper 2017/131. Helsinki, Finland: United Nations University; 2017. Available at https://scholar.harvard.edu/files/nunn/files/wp2017-131.pdf. Accessed 19 May 2020.

[18] Mugoya GCT, Witte TH, Ernst KC (2015). Sociocultural and victimization factors that impact attitudes toward intimate partner violence among Kenyan women. J Interpers Violence.

[19] Simwa SA. Secondary school students’ attitudes toward teaching and learning of feminist literature in Kenya. Adv Soc Sci Res J. 2017;4(9):21–8.

[20] Bennett L, Riger S, Schewe P, Howard A, Wasco S (2004). Effectiveness of hotline, advocacy, counseling, and shelter services for victims of domestic violence: a statewide evaluation. J Interpers Violence.

[21] Ullman SE (1996). Do social reactions to sexual assault victims vary by support provider?. Violence Vict.

[22] Wasco SM, Campbell R, Howard A (2004). A statewide evaluation of services provided to rape survivors. J Interpers Violence.

[23] Starzynski LL, Ullman SE, Filipas HH, Townsend SM (2005). Correlates of women's sexual assault disclosure to informal and formal support sources. Violence Vict.

[24] Bybee DI, Sullivan CM (2002). The process through which an advocacy intervention resulted in positive change for battered women over time. Am J Community Psychol.

[25] Sullivan CM (2003). Using the ESID model to reduce intimate male violence against women. Am J Community Psychol.

[26] Sullivan CM, Bybee DI (1999). Reducing violence using community-based advocacy for women with abusive partners. J Consult Clin Psychol.

[27] Hathaway JE, Willis G, Zimmer B (2002). Listening to survivors’ voices: addressing partner abuse in the health care setting. Violence Against Women.

[28] Logan TK, Evans L, Stevenson E, Jordan CE (2005). Barriers to services for rural and urban survivors of rape. J Interpers Violence..

[29] Decker MR, Wirtz AL, Baral SD (2012). Injection drug use, sexual risk, violence and STI/HIV among Moscow female sex workers. Sex Transm Infect.

[30] Glass N, Eden KB, Bloom T, Perrin N (2010). Computerized aid improves safety decision process for survivors of intimate partner violence. J Interpers Violence.

[31] Eden KB, Perrin NA, Hanson GC (2015). Use of online safety decision aid by abused women: effect on decisional conflict in a randomized controlled trial. Am J Prev Med.

[32] Glass N, Clough A, Case J (2015). A safety app to respond to dating violence for college women and their friends: the MyPlan study randomized controlled trial protocol. BMC Public Health.

[33] Campbell JC, Webster DW, Glass N (2009). The danger assessment:validation of a lethality risk assessment instrument for intimate partner Femicide. J Interpers Violence..

[34] Bandura A (1986). Social foundations of thought and action: a social cognitive theory.

[35] Dutton MA (1992). Empowering and healing the battered woman: a model for assessment and intervention.

[36] Substance Abuse and Mental Health Services Administration (SAMHSA) (2014). Trauma-Informed Care in Behavioral Health Services.

[37] Hopper EK, Bassuk EL, Olivet J (2010). Shelter from the storm: trauma-informed care in homeless service settings. Open Health Serv Policy J.

[38] Glass NE, Perrin NA, Hanson GC (2017). The longitudinal impact of an internet safety decision aid for abused women. Am J Prev Med.

[39] Bloom TL, Glass NE, Case J, Wright C, Nolte K, Parsons L (2014). Feasibility of an online safety planning intervention for rural and urban pregnant abused women. Nurs Res.

[40] Koziol-McLain J, Vandal AC, Nada-Raja S (2015). A web-based intervention for abused women: the New Zealand isafe randomised controlled trial protocol. BMC Public Health.

[41] Guest G, Bunce A, Johnson L (2006). How many interviews are enough? An experiment with data saturation and variability. Field Methods.

[42] Farmer T, Robinson K, Elliott SJ, Eyles J (2006). Developing and implementing a triangulation protocol for qualitative health research. Qual Health Res.

[43] Ethical and safety recommendations for intervention research on violence against women. Building on lessons from the WHO publication Putting women first: ethical and safety recommendations for research on domestic violence against women. Geneva: World Health Organization; 2016.

[44] O'Connor AM (1995). Validation of a decisional conflict scale. Med Decis Mak.

[45] McFarlane J, Parker B, Soeken K, Silva C, Reel S (1998). Safety behaviors of abused women after an intervention during pregnancy. J Obstet Gynecol Neonatal Nurs.

[46] STRAUS MA, HAMBY SL, BONEY-McCOY S, SUGARMAN DB (1996). The revised conflict tactics scales (CTS2):development and preliminary psychometric data. J Fam Issues.

[47] Smith PH, Earp JA, DeVellis R (1995). Measuring battering: development of the Women's Experience with Battering (WEB) Scale. Women's health (Hillsdale, NJ).

[48] McCauley HL, Silverman JG, Jones KA (2017). Psychometric properties and refinement of the reproductive coercion scale. Contraception..

[49] Miller E, Decker MR, McCauley HL (2011). A family planning clinic partner violence intervention to reduce risk associated with reproductive coercion. Contraception..

[50] Campbell-Sills L, Stein MB (2007). Psychometric analysis and refinement of the connor–Davidson resilience scale (CD-RISC): validation of a 10-item measure of resilience. J Trauma Stress.

[51] Klasen F, Oettingen G, Daniels J, Post M, Hoyer C, Adam H (2010). Posttraumatic resilience in former Ugandan child soldiers. Child Dev.

[52] Andresen EM, Malmgren JA, Carter WB, Patrick DL (1994). Screening for depression in well older adults: evaluation of a short form of the CES-D (Center for Epidemiologic Studies Depression Scale). Am J Prev Med.

[53] Radloff LS (1977). The CES-D scale:a self-report depression scale for research in the general population. Appl Psychol Meas.

[54] Breitenbecher KH (2006). The relationships among self-blame, psychological distress, and sexual victimization. J Interpers Violence.

[55] Rothman EF, Decker MR, Silverman JG (2006). Evaluation of a teen dating violence social marketing campaign: lessons learned when the null hypothesis was accepted. N Dir Eval.

[56] R S, M J (1995). Generalized self-efficacy scale.

[57] Dupont WD, Plummer WD (1998). Power and sample size calculations for studies involving linear regression. Control Clin Trials.

[58] Anderson EJ, Krause KC, Meyer Krause C, et al. Web-based and mHealth interventions for intimate partner violence victimization prevention: a systematic review. Trauma Violence Abuse. 2019;1524838019888889. 10.1177/1524838019888889 31742475

[59] Kok MC, Dieleman M, Taegtmeyer M (2015). Which intervention design factors influence performance of community health workers in low- and middle-income countries? A systematic review. Health Policy Plan.

[60] Wendell DA, Cohen DA, LeSage D, Farley TA (2003). Street outreach for HIV prevention: effectiveness of a state-wide programme. Int J STD AIDS.

[61] Mock J, McPhee SJ, Nguyen T (2007). Effective lay health worker outreach and media-based education for promoting cervical cancer screening among Vietnamese American women. Am J Public Health.

[62] Paskett E, Tatum C, Rushing J (2006). Randomized trial of an intervention to improve mammography utilization among a triracial rural population of women. J Natl Cancer Inst.

[63] Katz ML, Tatum CM, Degraffinreid CR, Dickinson S, Paskett ED (2007). Do cervical cancer screening rates increase in association with an intervention designed to increase mammography usage?. J Women's Health (Larchmt).

[64] Viswanathan M, Kraschnewski JL, Nishikawa B (2010). Outcomes and costs of community health worker interventions: a systematic review. Med Care.

[65] Gary TL, Bone LR, Hill MN (2003). Randomized controlled trial of the effects of nurse case manager and community health worker interventions on risk factors for diabetes-related complications in urban African Americans. Prev Med.

[66] Krieger JW, Takaro TK, Song L, Weaver M (2005). The Seattle-King County healthy homes project: a randomized, controlled trial of a community health worker intervention to decrease exposure to indoor asthma triggers. Am J Public Health.

[67] Braun R, Catalani C, Wimbush J, Israelski D (2013). Community health workers and mobile technology: a systematic review of the literature. PLoS One.

[68] Bakibinga P, Kamande E, Omuya M, Ziraba AK, Kyobutungi C (2017). The role of a decision-support smartphone application in enhancing community health volunteers’ effectiveness to improve maternal and newborn outcomes in Nairobi, Kenya: quasi-experimental research protocol. BMJ Open.

